# Angelica sinensis polysaccharide inhibits pyroptosis and improves osteoarthritis through the collaborative power of PPARγ and Nrf2

**DOI:** 10.3389/fphar.2026.1844591

**Published:** 2026-08-06

**Authors:** Ning Yi, Long-Xin Yu, Hang Yuan, Zhuang-Zhuang Zhang, Su Ni, Chao Zhuang

**Affiliations:** 1 Department of Orthopedics, Liaoning Fangda General Hospital, Shenyang, China; 2 Department of Orthopedics, The Second People’s Hospital of Changzhou, The Third Affiliated Hospital of Nanjing Medical University, Changzhou, China; 3 Department of Orthopedics, People’s Hospital of Quzhou, Quzhou, China

**Keywords:** angelica sinensis polysaccharide, Nrf2, osteoarthritis, PPARγ, pyroptosis

## Abstract

**Background and aim:**

Osteoarthritis (OA) is an inflammatory disease, and pyroptosis causes a local inflammatory microenvironment that leads to chondrocyte disorder. This article aims to show that angelica sinensis polysaccharide (ASP) can improve the antioxidant and anti-inflammatory capacity of chondrocytes through the collaborative power of PPARγ and Nrf2, downregulate pyroptosis, and thus alleviate OA.

**Methods:**

After analyzing the clinical samples, the therapeutic effect of ASP was assessed *in vitro* and *in vivo*, respectively. The concentrations of lipopolysaccharide (LPS) with ATP were determined by CCK-8. Pyroptosis was evaluated by lactate dehydrogenase (LDH) release, caspase-1 staining, cell viability and cytotoxicity, mitochondrial membrane potential (MMP) detection, and measurement of ROS. The expression of PPARγ, Nrf2, NLRP3, ASC, caspase-1, GSDMD-N, NF-κB, SOD2, and Cyt-C was detected by qRT-PCR, Western blot, immunofluorescence, and immunohistochemistry.

**Results:**

The expression of the NLRP3 inflammasome in OA cartilage was increased, and inflammatory cytokines were higher in OA serum. LPS promoted oxidative stress and induced pyroptosis in chondrocytes, while ASP mediated the collaborative power of PPARγ and Nrf2 to improve the viability of chondrocytes, reduce the activity of the NLRP3 inflammasome, and inhibit pyroptosis.

**Conclusion:**

These findings revealed the process of pyroptosis in chondrocytes and the enhancement of PPARγ and Nrf2 by ASP, which deepens our understanding of OA pathogenesis and could provide new insights for treatment.

## 1 Introduction

Osteoarthritis (OA) is an inflammatory joint disease with degenerative articular cartilage and synovitis as the main pathological changes. Chondrocytes are the only cell type in articular cartilage (O'Neill and Felson, 2018; Chen et al., 2017; Pereira et al., 2011). Chondrocytes can maintain the stability of the extracellular matrix (ECM) under physiological conditions. However, under pathological conditions, the internal environment of the joint cavity changes, breaking the metabolic balance of chondrocytes, followed by structural disorder of chondrocytes, degradation of ECM, and subchondral osteosclerosis or osteoporosis (Abra et al., 2020; Be and renbaum, 2013).

Pyroptosis, also known as inflammatory necrosis, is a kind of programmed cell death characterized by the release of inflammatory cytokines, which is different from other types of cell death (Lu et al., 2020). Pyroptosis is an important immune response for body defense, which plays a crucial role in resisting infection and activating endogenous danger signals (Latz et al., 2013). When an inflammasome such as NLRP3 senses infection or inherent danger signals, it activates caspase-1 by binding to apoptosis-associated speck-like protein containing a CARD (ASC), and then caspase-1 cleaves GSDMD, inducing canonical pyroptosis (Shi et al., 2015; Kovacs et al., 2017).

Peroxisome proliferator-activated receptor gamma (PPARγ) is a nuclear transcription factor that plays an important role in the cell cycle, inflammatory response, and ECM metabolism (Yuan et al., 2024). Nuclear factor erythroid 2-related factor 2 (Nrf2) is a major transcription factor regulating endogenous antioxidant defense, which can drive the transcription of multiple genes regulated by antioxidant response elements (Chen et al., 2019). These genes are involved in crucial cell life processes, including redox regulation, protein homeostasis, exogenous detoxification, and material metabolism (Lee, 2017).

Angelica sinensis polysaccharide (ASP) is the main water-soluble component of *Angelica sinensis*, a traditional Chinese medicine, with a wide range of pharmacological activities such as improve blood circulation and disperse stasis, anti-inflammatory, antioxidant, immune regulation, etc. (Cao et al., 2010; Ai et al., 2013; Yu et al., 2013; Zhang et al., 2010; Hua et al., 2014; Liu et al., 2010; Sun et al., 2005). Our previous studies have shown that ASP has both *in vitro* and *in vivo* antioxidant and anti-inflammatory activities that can inhibit chondrocyte apoptosis and alleviate the degeneration of OA cartilage (Ni et al., 2023; Zhuang et al., 2018; Zhuang et al., 2020; Zhuang et al., 2016). These findings suggest that ASP is a potential natural therapeutic drug for OA, but its specific mechanism has not been thoroughly studied.

In this article, we speculated that the local inflammatory microenvironment of OA induced by pyroptosis was the key that led to cartilage degeneration in OA. ASP could improve the viability of chondrocytes through PPARγ collaborating with Nrf2, regulate the activation of the NLRP3 inflammasome, reduce the release of inflammatory cytokines, and thus alleviate the progression of OA.

## 2 Materials and methods

### 2.1 Reagents

ASP (Supplementary Figure S1A) (CAT.B25568) was purchased from Yuanye Bio-Technology Co., Ltd (Shanghai, China) and analyzed using an UV spectrophotometer to scan the spectrum at full wavelength. As shown in Supplementary Figure S1B, there was no obvious characteristic absorption peak between 280 nm and 290 nm, indicating that the protein had been basically removed. The polysaccharides were dissolved in deionized water to a concentration of 30 mg/mL. After centrifugation at 4000 rpm for 10 min, 3 mL of supernatant was loaded onto a DEAE-cellulose-52 column (2.6 × 40 cm) that had been equilibrated with distilled water. The column was eluted with a stepwise gradient with 0, 0.1 M, 0.2 M, and 0.4 M NaCl solutions at a flow rate of 2 mL/min, and subsequently the fractions were collected based on the phenol-sulfuric acid procedure. The monosugar compositions of ASP were mannose: rhamnose: glucose: galactose: arabinose = 1.15:1.76:1.84:1.82:0.84 (Supplementary Figure S1C). The absorbance of ASP detected by the phenol-sulfuric acid method was 0.500, and the polysaccharide purity calculated from the standard curve was 97.2%, with an average molecular weight of 85.0 kDa.

ADTC5 cells were obtained from Zhong Qiao Xin Zhou Biotechnology Co., Ltd. (Shanghai, China). DMEM supplied with 100 U penicillin, 100 μg streptomycin, and 10% FBS was obtained from Gibco (Grand Island, NY, United States). The lipopolysaccharide (LPS), ATP, cell counting kit-8 (CCK-8), LDH assay kit, cell viability/cytotoxicity assay kit, caspase-1 assay kit, mitochondrial membrane potential (MMP) assay kit, and SOD2 assay kit were purchased from Beyotime Biotechnology (Shanghai, China). MitoSOX Red mitochondrial superoxide indicator was obtained from Yeasen Biotechnology (Shanghai, China). ELISA kits for IL-1β and IL-18 and TRIzol were obtained from Invitrogen (Carlsbad, CA, United States). The high-capacity cDNA reverse transcription kit was obtained from Applied Biosystems (Foster City, CA, United States). SYBR® Select Master Mix was obtained from Applied Biosystems (Austin, TX, United States).

ASP was purchased from Yuanye Bio-Technology Co., Ltd (Shanghai, China) and analyzed by UV spectrophotometer. The purity was 97.2%, and the average molecular weight was 85.0 kDa. It was dissolved in DMSO. GW1929, NK-252, N-acetylcysteine (NAC), and MCC950 were purchased from MedChemExpress (Shanghai, China) and dissolved in DMSO. HE and safranin O/fast green staining kits were purchased from Solarbio (Beijing, China).

### 2.2 Clinical samples

Serum samples were obtained from 30 OA patients who were diagnosed according to the 1985 criteria of the American Rheumatism Association and underwent total knee replacement (Kellgren–Lawrence (KL) score 3–4). Among these, six samples of relatively normal cartilage and damaged cartilage were gathered for pathological examination and Western blot. Sections were stained with hematoxylin and eosin (HE) as well as safranin O/fast green according to the manufacturer’s recommendations. To determine the extent of cartilage degeneration, histological scoring methods issued by Mankin and the Osteoarthritis Research Society International (OARSI) were used for analysis. A total of 40 serum samples of healthy individuals were included as normal controls. This study was reviewed and approved by the ethics committee of the Second People’s Hospital of Changzhou, the Third Affiliated Hospital of Nanjing Medical University, Jiangsu, China ([2023] KY222-01).

### 2.3 ELISA

Serum IL-1β and IL-18 detection were carried out according to the manufacturer’s instructions. In brief, the precoated plate was brought to room temperature. Specimens (100 μL) or standard substances with different concentrations were added to the corresponding wells and incubated in a water bath kettle at 37 °C for 90 min. To each well was added 100 μL biotinylated antibody working solution, and the plate was incubated at 37 °C for 60 min. After washing, 100 μL enzyme conjugate working solution was added to each well, and the plate was incubated at 37 °C for 30 min in the dark. Then, 100 μL of TMB color substrate was added, and the plate was incubated at 37 °C for 15 min in the dark. After adding 100 μL reaction stop solution, absorbance at 450 nm was measured immediately using a microplate reader (Elx808™ Bio-Tek Instruments, Winooski, VT, United States).

### 2.4 Cell proliferation

To assess the concentration of LPS *in vitro*, cell proliferation was evaluated using the CCK-8 assay. ADTC5 cells were plated in 96-well plates at a density of 5 × 10^3^ cells/well to adhere overnight and treated with LPS (1 μg/mL, 2 μg/mL, 3 μg/mL, 4 μg/mL, 5 μg/mL, and 6 μg/mL) and 5 μg/mL LPS with ATP (0.5 mol/mL, 1 mol/mL, 1.5 mol/mL, 2.0 mol/mL, 2.5 mol/mL, and 3.0 mol/mL) for 3 h, 6 h, 12 h, and 24 h. After incubation, 10 μL of CCK-8 solution was added to each well and further incubated for 1 h at 37 °C in 5% CO_2_. Absorbance at 450 nm was measured using a microplate reader (Elx808™ Bio-Tek Instruments, Winooski, VT, United States).

### 2.5 Cell treatment

ADTC5 cells were pretreated with ASP (100 μg/mL) or GW1929 (10 μmol/mL) or NK-252 (5 μmol/mL) or NAC (5 μmol/mL) or MCC950 (10 μmol/mL) for 2 h and then treated with 5 μg/mL LPS and 2.0 mol/mL ATP for 12 h. The following experiments were conducted according to the routine procedures.

### 2.6 Caspase-1/propidium iodide double staining

For pyroptosis analysis, active caspase-1 and propidium iodide (PI) fluorescence of samples were measured according to the manufacturer’s instructions. In brief, the cell density was adjusted to 5 × 10^5^/mL, and the working solution was added to the cell suspension at a ratio of 1:30–1:60 (v/v). The cells were incubated at 37 °C for 45 min and centrifuged at 12,000 rpm for 5 min at room temperature. Next, the cell supernatants were discarded, and the cells were resuspended in wash buffer. Then, the PI was added to the cell suspension 5 min prior to analysis using flow cytometry (BD Biosciences, San Jose, CA, United States).

### 2.7 LDH release

ADTC5 cells were plated in a 96-well plate at a density of 5 × 10^3^ cells/well to adhere overnight. After treatment, the levels of LDH were determined using an assay kit with a microplate reader (Elx808™ Bio-Tek Instruments, Winooski, VT, United States) following the manufacturer’s instructions.

### 2.8 Cell viability and cytotoxicity

ADTC5 cells were washed with PBS once, and calcein AM/PI test working fluid was added. The plate was incubated in the dark at 37 °C for 30 min, then the calcein AM green (excitation 494 nm/emission 517 nm) and PI red (excitation 535 nm/emission 617 nm) fluorescence was observed by fluorescence microscope (CX41-32RFL, OLYMPUS, Tokyo, Japan).

### 2.9 MMP detection

JC-1 probe was employed to measure mitochondrial depolarization. In brief, ADTC5 cells were incubated with an equal volume of JC-1 staining solution at 37 °C for 20 min and rinsed twice with PBS. MMP values were monitored by determining the relative amounts of dual emissions from mitochondrial JC-1 monomers or aggregates using a fluorescence microscope (CX41-32RFL, OLYMPUS, Tokyo, Japan).

### 2.10 Measurement of ROS

The level of ROS was measured according to the manufacturer’s instructions. ADTC5 cells were incubated with 5 μM MitoSOX reagent working solution for 10 min at 37 °C in the dark. After washing three times with warm HBSS, ROS intensity was detected with a fluorescent microscope (CX41-32RFL, OLYMPUS, Tokyo, Japan).

### 2.11 qRT-PCR

Total RNA was extracted using TRIzol, and a high-capacity cDNA reverse transcription kit was used to reverse transcribe total RNA. PPARγ, Nrf2, NLRP3, ASC, caspase-1, GSDMD-N, NF-κB, and GAPDH were amplified using SYBR® Select Master Mix in a Bio-Rad iQ5. The specific primer sequences are presented in Table 1. The data were calculated by the comparative threshold cycle method.

**TABLE 1:** Primer sequences for qRT-PCR.

Gene	Forward (5'→3')	Reverse (5'→3')
*PPARγ*	TCG​CTG​ATG​CAC​TGC​CTA​TG	GAG​AGG​TCC​ACA​GAG​CTG​ATT
*Nrf2*	ACC​AAG​GGG​CAC​CAT​ATA​AAA​G	CTT​CGC​CGA​GTT​GCA​CTC​A
*NLRP3*	ATTACCCGCCCGAGAAAGG	TCGCAGCAAAGATCCACACAG
*ASC*	AGGTGGACTGGATACACAGAC	TCTCCTGCCCAAACTCTTTGC
caspase-1	ACAAGGCACGGGACCTATG	TCCCAGTCAGTCCTGGAAATG
GSDMD-N	CCATCGGCCTTTGAGAAAGTG	ACACATGAATAACGGGGTTTCC
NF-κB	TGCCCCTGTGGTCAAAGTG	GGTTCGGTTACCGTCCTGC
*GAPDH*	TGG​ATT​TGG​ACG​CAT​TGG​TC	TTT​GCA​CTG​GTA​CGT​GTT​GAT

### 2.12 Western blot

Human cartilage samples were subjected to additional grinding in liquid nitrogen. ADTC5 cells were harvested and lysed in RIPA buffer for total protein extraction. Equal amounts of protein were separated by SDS-PAGE and transferred onto PVDF membrane. After being blocked for 1 h in Tris-buffered saline with Tween-20 with 5% nonfat milk, the PVDF membrane was then probed with human and mouse primary antibodies (Cell Signaling Technology, Danvers, MA, United States; and diluted 1:1000) overnight at 4 °C and then with horseradish peroxidase (HRP) conjugated secondary antibody (Cell Signaling Technology, Danvers, MA, United States; and diluted in 1:5,000) for 1 h at room temperature. The blots were detected using the enhanced chemiluminescence assay. The β-actin signal was used as an internal loading control, and relative expression levels were quantified using Quantity One software (Bio-Rad Laboratories, Hercules, CA, United States).

### 2.13 Immunofluorescence

ADTC5 cells were seeded on the gelatin-pre-coated slices and grown to 50%–60% confluence. Then, the slices were fixed with 4% formaldehyde for 30 min, penetrated with 0.5% Triton X-100 for 10 min, and blocked with 1% BSA for 1 h at room temperature. Rabbit anti-mouse primary antibodies (Cell Signaling Technology, Danvers, MA, United States) diluted 1:1000 were added and incubated in a wet box at 4 °C overnight. The fluorescent second antibody was added, incubated in a wet box, and placed at room temperature for 1 h. Sealed slices were stored in a refrigerator at −20 °C and photographed under a fluorescence microscope (CX41-32RFL, OLYMPUS, Tokyo, Japan).

### 2.14 Cell transfection

ADTC5 cells were seeded at 1 × 10^6^ cells/ml in 6-well plates and cultured for 24 h before experimentation. PPARγ siRNA, Nrf2 siRNA, and normal cartilage (NC) siRNA were produced by RiboBio (Guangzhou, China). The PPARγ-specific siRNA sequence was 5′-CAA​CAG​GCC​UCA​UGA​AGA​A-3′, the Nrf2-specific siRNA sequence was 5′-GCA​UGA​UGG​ACU​UGG​AGU​U-3′, and the non-targeting control was 5′-UAG​CGA​CUA​AAC​ACA​UCA​A-3′. The siRNA (100 nM) was transfected into ADTC5 cells by Lipofectamine RNAiMAX for 6 h according to the manufacturer’s instructions. Then, ADTC5 cells were cultured in DMEM for another 36 h.

### 2.15 SOD2 activity

The enzyme activity of SOD2 was determined using an assay kit according to the manufacturer’s instructions. Briefly, SOD-1 inhibitors A and B were successively added to the samples to inhibit residual SOD-1 activity for 60 min at 37 °C and then mixed with WST-8 enzyme working solution for 30 min at 37 °C. The absorbance at 450 nm was measured using a microplate reader (Elx808™ Bio-Tek Instruments, Winooski, VT, United States).

### 2.16 Animal experimentation

Thirty 6-week-old male C57BL/6 mice (Slac Laboratory Animal Co. Ltd, Shanghai, China) were randomly divided into six groups: Sham, destabilization of the medial meniscus (DMM), ASP, GW1929, NK-252, and NAC. Destabilization of the medial meniscus (DMM) was undertaken to construct an osteoarthritis model in the right knee (Culley et al., 2015). In brief, after abdominal anesthesia with chloral hydrate and routine skin preparation, a longitudinal incision was made on the medial part of the knee, and the medial meniscotibial ligament was exposed and transected to destabilize the medial meniscus. The sham surgery was also performed by a similar surgical approach without manipulating the joint tissue. Four weeks after surgery, the drug groups were intragastrically administered with a 5 mg/kg dose, twice a week. Four weeks after administration, the mice were sacrificed, and the right hind limbs were collected for analysis. The protocol was approved by the Institutional Animal Care and Use Committee of Nanjing Medical University (IACUC-2407014).

### 2.17 X-ray examination

Two right hind limbs of the mice from each group were collected post-anesthesia, with soft tissues removed, and fixed in 4% paraformaldehyde solution. The right hind limbs were subjected to lateral views by X-ray.

### 2.18 Immunohistochemistry

The specimens were fixed overnight in 4% paraformaldehyde, decalcified in 10% EDTA, followed by embedding in paraffin and sectioning. Sections were dewaxed in xylene, dehydrated in graded alcohol, and incubated with 3% peroxide-methanol at room temperature to block endogenous peroxidases for 10 min. After the microwave retrieval and wash, the nonspecific staining was blocked by incubating the sections with bovine serum albumin for 30 min at 37 °C. The primary antibodies were incubated overnight at 4 °C. On the following day, the sections were washed and incubated with the secondary antibody for 40 min at 37 °C, followed by washing and incubating with the horseradish-labeled avidin for 40 min at 37 °C. Finally, the sections were stained with diaminobenzidine until the cytoplasm was stained in brown.

### 2.19 Statistical analysis

Data shown in our study are presented as the means ± SD from a minimum of three independent experiments. One-way ANOVA, followed by the Bonferroni test, was conducted for comparisons between two groups. The difference was considered significant when *P* < 0.05.

## 3 Results

### 3.1 Bioinformatics analysis

**FIGURE 1:**
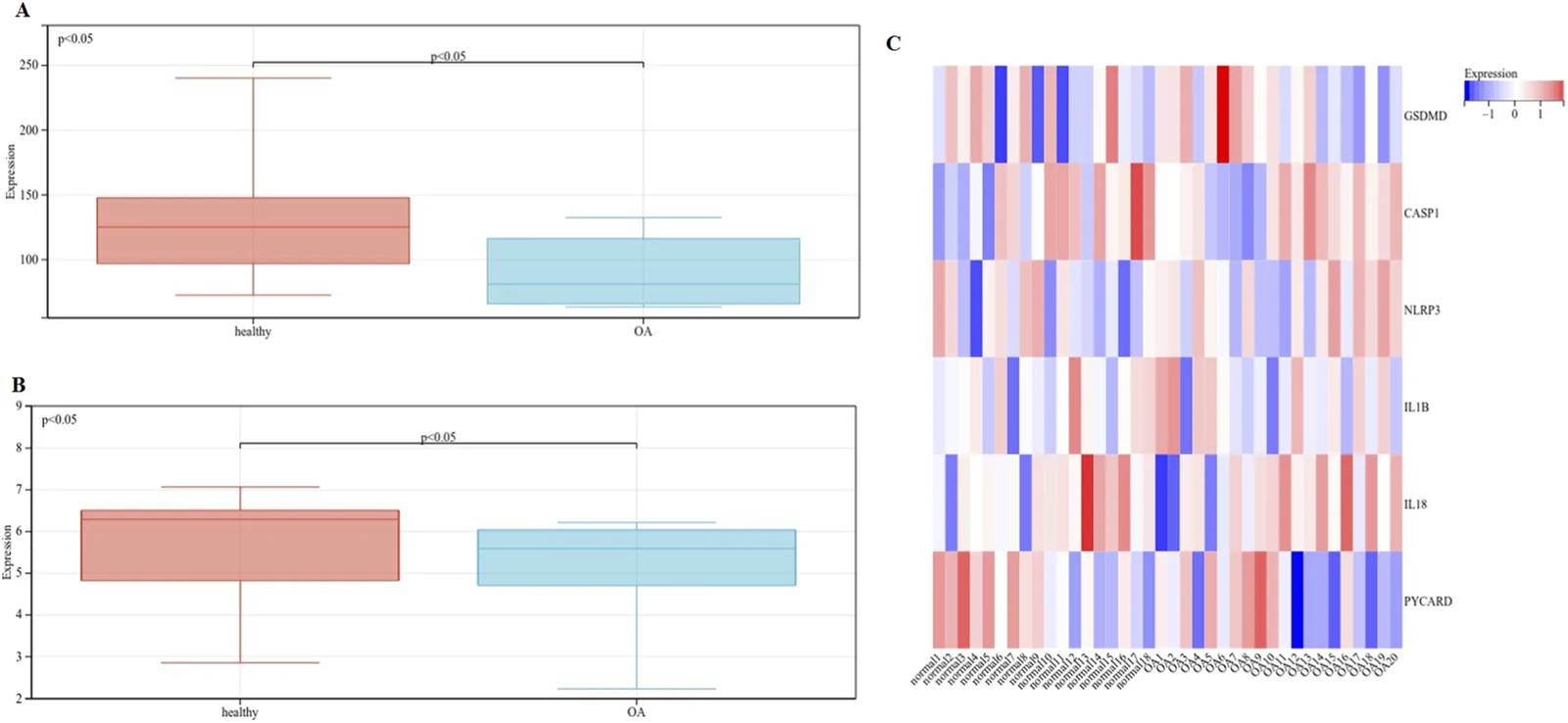
Bioinformatics analysis. Box plots show the downregulation of PPARγ **(A)** and Nrf2 **(B)** expressions in OA cartilage (*p* < 0.05). **(C)** Heatmap shows the upregulation of pyroptosis-related genes in OA cartilage.

The OA-related chip datasets GSE114007 and GSE55235 were obtained from the NCBI GEO database. All datasets contain whole-genome expression data and corresponding sample annotation information for the OA group and the normal control group. The downloaded original expression matrix was preprocessed uniformly, standardized corrections were applied, and targeted gene expression analysis was conducted based on this. The expression level data of the PPARγ gene were extracted from the GSE55235 dataset, a box plot was constructed, and the expression differences between the OA group and the normal control group were compared. The results indicate a statistically significant difference (Figure 1A). For the GSE114007 dataset, the expression level data of the Nrf2 gene in the OA group and the normal control group were extracted, and a box plot was drawn to compare the expression difference of this gene in the two groups. The test results indicate statistical significance (Figure 1B). Based on the GSE114007 dataset, pyroptosis-related genes were screened, and expression level data were extracted. A heat map was drawn to visually present the expression difference patterns and clustering features between the two groups (Figure 1C).

### 3.2 Histological characteristics of OA

**FIGURE 2:**
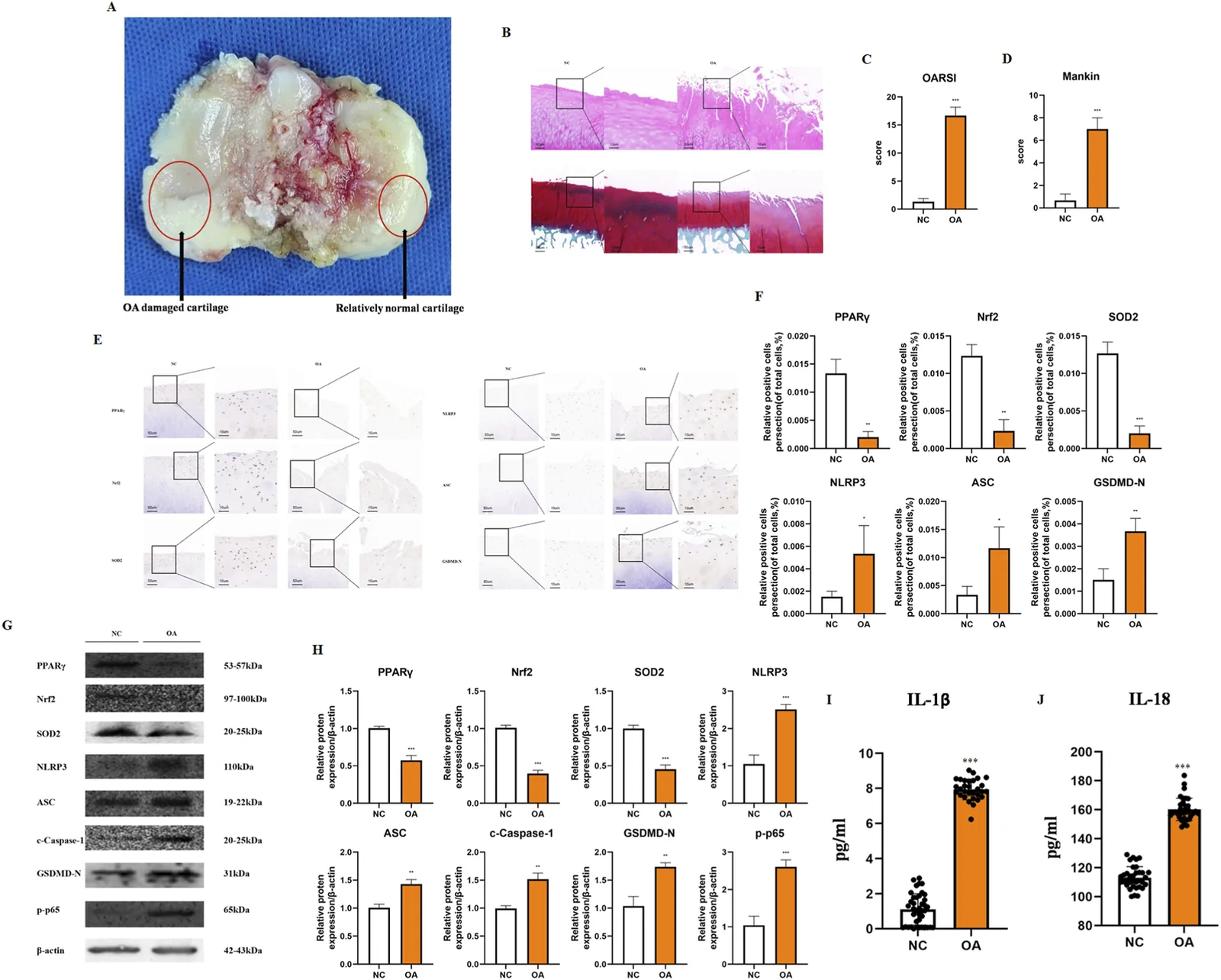
Histological characteristics of OA. **(A)** Specimen of OA cartilage tissue. **(B)** Images of HE and safranin O/fast green staining (×5 magnification, scale bar, 10 μm). **(C)** OARSI score. **(D)** Mankin score. **(E)** Immunohistochemistry of relative proteins (×5 magnification, scale bar, 10 μm). **(F)** Quantification data of immunohistochemistry. **(G)** Western blot of relative proteins. **(H)** WB quantification data. ELISA of serum IL-1β **(I)** and IL-18 **(J)**. *p < 0.05, **p < 0.01, ***p < 0.001 versus NC.

The cartilage of the NC group had a smooth surface, orderly arrangement of chondrocytes, and normal safranin staining, while the OA group showed damaged surface structure, reduced chondrocytes, fiber proliferation, and decreased safranin staining (Figure 2B). Compared with the NC group, the OARSI score and Mankin score of the OA group were significantly increased (Figures 2C,D). Immunohistochemistry showed downregulation of PPARγ and Nrf2 in OA cartilage tissue. SOD2, as a secondary antioxidant enzyme, is a common target gene of PPARγ and Nrf2 and undergoes transcriptional activation, resulting in lighter staining, which was consistent with other studies. NLRP3, ASC, and GSDMD-N are characteristic protein markers of pyroptosis, and all three were significantly elevated in OA cartilage tissue, indicating that pyroptosis plays a key role in OA inflammation (Figures 2E,F).

Further grinding of cartilage tissue and Western blot (WB) detection showed an increase in p-p65 expression, suggesting the activation of the classical NF-κB signaling pathway, which led to the hydrolysis of caspase-1. Activated caspase-1 forms an inflammasome with NLRP3 and ASC, activating the classical pyroptosis pathway dependent on caspase-1, thereby hydrolyzing GSDMD and increasing GSDMD-N expression, consistent with immunohistochemical results (Figures 2G,H). ELISA detection also showed that the serum levels of IL-1β and IL-18 in OA patients were higher than those in the NC group, indicating that pyroptosis is involved in the pathogenesis of OA (Figures 2I,J).

### 3.3 Inducing pyroptosis of chondrocytes

**FIGURE 3:**
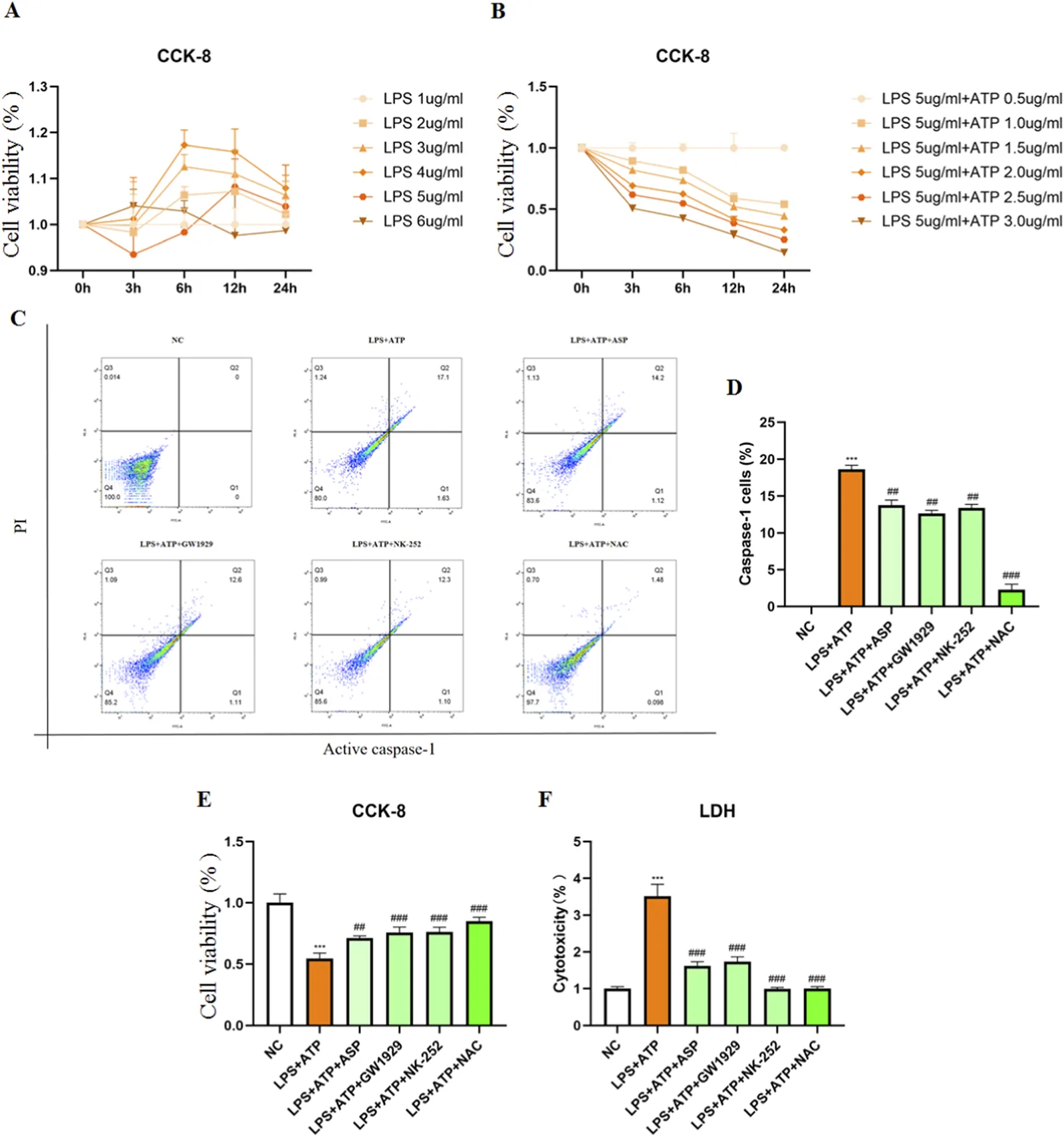
Inducing pyroptosis of chondrocytes. **(A)** Pyroptosis induced with LPS. **(B)** Pyroptosis induced with LPS + ATP. **(C)** Detection of active caspase-1 by flow cytometry. **(D)** Flow cytometry quantification data. **(E)** CCK-8 detection. **(F)** LDH detection. NC was used as a control and considered 100% viable.***p < 0.001 versus NC. ^##^ *p* < 0.01, ^###^ *p* < 0.001 versus LPS + ATP.

To induce pyroptosis in ADTC5 cells, we first intervened with different concentrations of LPS. CCK-8 detection showed that the effect of LPS alone was unstable (Figure 3A). Therefore, we added different concentrations of ATP with LPS and ultimately chose 5 μg/mL LPS+2.0 mol/mL ATP as the stimulus for inducing pyroptosis (Figure 3B). After successfully establishing a model of pyroptosis, we selected GW1929 and NK-252, which are chemical agonists of PPARγ and Nrf2, as intervention controls. NAC, a common antioxidant, served as the positive control for ASP. Flow cytometry showed a significant increase in activated caspase-1 in the LPS + ATP group (Figures 3C,D), indicating continued progression of pyroptosis. The CCK-8 assay showed a significant decrease in cell viability in the LPS + ATP group (Figure 3E), and LDH detection indicated a significantly higher level than in the NC group (Figure 3F), suggesting that pyroptosis led to increased cell toxicity. These cellular experimental results were consistent with the detection of clinical specimens from OA patients.

Pretreatment with ASP effectively inhibited the activated caspase-1 level, enhanced cell viability, and reduced LDH release, thus alleviating cytotoxicity. The effect was comparable to that of the NAC group and was also consistent with the GW1929 and NK-252 groups (Figures 3C–F), which suggested that PPARγ and Nrf2 were involved in pyroptosis, and that the downregulation of pyroptosis by ASP might be related to PPARγ and Nrf2.

### 3.4 Pyroptosis led to mitochondrial dysfunction

**FIGURE 4:**
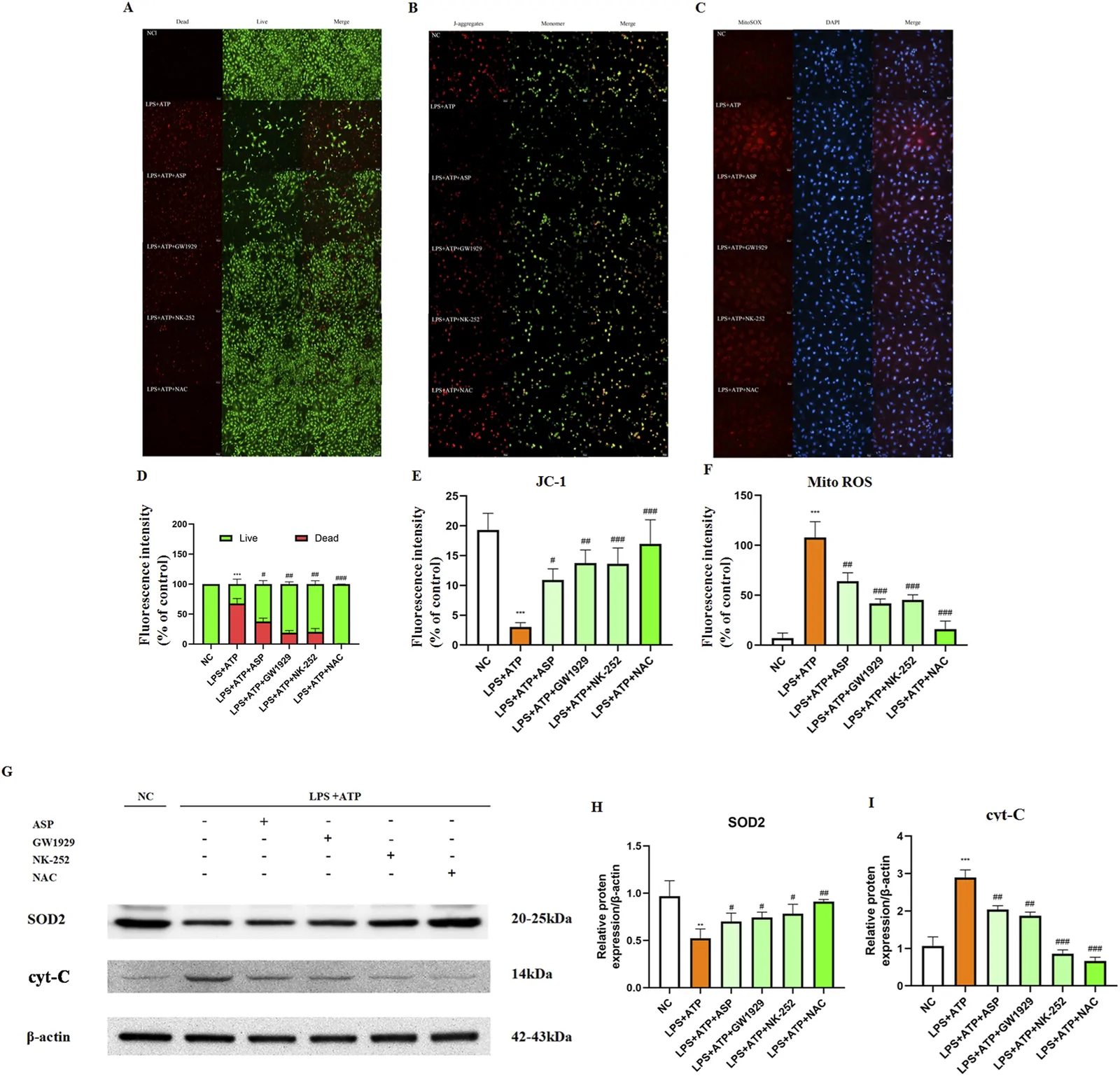
Pyroptosis led to mitochondrial dysfunction. **(A)** Cell viability and cytotoxicity. **(B)** JC-1 staining of MMP. **(C)** Fluorescence of mitochondrial ROS. **(D-F)** Fluorescence intensity quantification data. **(G)** Western blot of SOD2 and Cyt-C. **(H)** SOD2 quantification data. **(I)** Cyt-C quantification data. **p < 0.01, ***p < 0.001 versus NC. ^#^ *p* < 0.05, ^##^ *p* < 0.01, ^###^ *p* < 0.001 versus LPS + ATP.

Pyroptosis is a process involving the release of inflammatory mediators, which is consistent with the course of aseptic inflammation in OA. During this process, the internal environment of chondrocytes changes. The results of cell viability and cytotoxicity assays showed that the number of viable cells in the LPS + ATP group was significantly decreased compared to the NC group (Figures 4A,D). Cell viability is closely related to mitochondrial function. JC-1 staining revealed that the MMP in the LPS + ATP group was significantly lower than that in the NC group (Figures 4B,E), indicating increased mitochondrial permeability, impaired structural integrity of mitochondria, and mitochondrial dysfunction. WB indicated that the leakage of Cyt-C in the LPS + ATP group was significantly increased compared to the NC group (Figures 4G,I), thereby activating the endogenous pathways of apoptosis and leading to an increase in the number of dead cells in the LPS + ATP group. Furthermore, persistent inflammation could lead to oxidative stress within chondrocytes, while the activity of SOD2 in the LPS + ATP group was significantly decreased (Figures 4G,H), preventing timely clearance of mitochondrial ROS. The red fluorescence intensity in the LPS + ATP group was significantly higher than that in the NC group (Figures 4C,F), indicating further exacerbation of mitochondrial dysfunction and forming a vicious cycle. After ASP pretreatment, the expression of SOD2 could be significantly increased, eliminating mitochondrial ROS, stabilizing MMP, reducing Cyt-C leakage, restoring chondrocyte activity, and thereby reversing the pyroptosis effects. SOD2 is a downstream target gene shared by both PPARγ and Nrf2. ASP showed an upregulation of SOD2 similar to that of GW1929 or NK-252, suggesting that ASP’s alleviating effect on mitochondrial dysfunction induced by pyroptosis was related to PPARγ and Nrf2.

### 3.5 Pyroptosis interfered with the expressions of PPARγ and Nrf2 in chondrocytes

In the detection of OA clinical specimens, we showed that the markers of pyroptosis showed an upward trend, while the protein expressions of PPARγ and Nrf2 were downregulated. After successfully inducing pyroptosis using LPS + ATP, we further corroborated the above conclusion through *in vitro* experiments using qRT-PCR and WB. The results showed that PPARγ and Nrf2 in chondrocytes stimulated by LPS + ATP decreased significantly at both the gene and protein levels, whereas NLRP3, ASC, and c-caspase-1 exhibited the opposite trend, with all three significantly increasing. This led to the formation of an inflammasome, which hydrolyzed GSDMD and promoted the release of inflammatory mediators. Simultaneously, the NF-κB signaling pathway was also activated, and its relationship with pyroptosis remains to be verified. After ASP pretreatment, the gene and protein expressions of PPARγ and Nrf2 were restored, and the expressions of pyroptosis-related markers decreased, further indicating that ASP’s inhibitory effect on the NLRP3 inflammasome was associated with PPARγ and Nrf2. Meanwhile, the results also showed that GW1929 had an upregulating effect on Nrf2, as well as NK-252 on PPARγ. Based on this, we suggested a synergistic promoting effect between PPARγ and Nrf2.

### 3.6 PPARγ and Nrf2 interact to inhibit mitochondrial ROS generation

**FIGURE 5:**
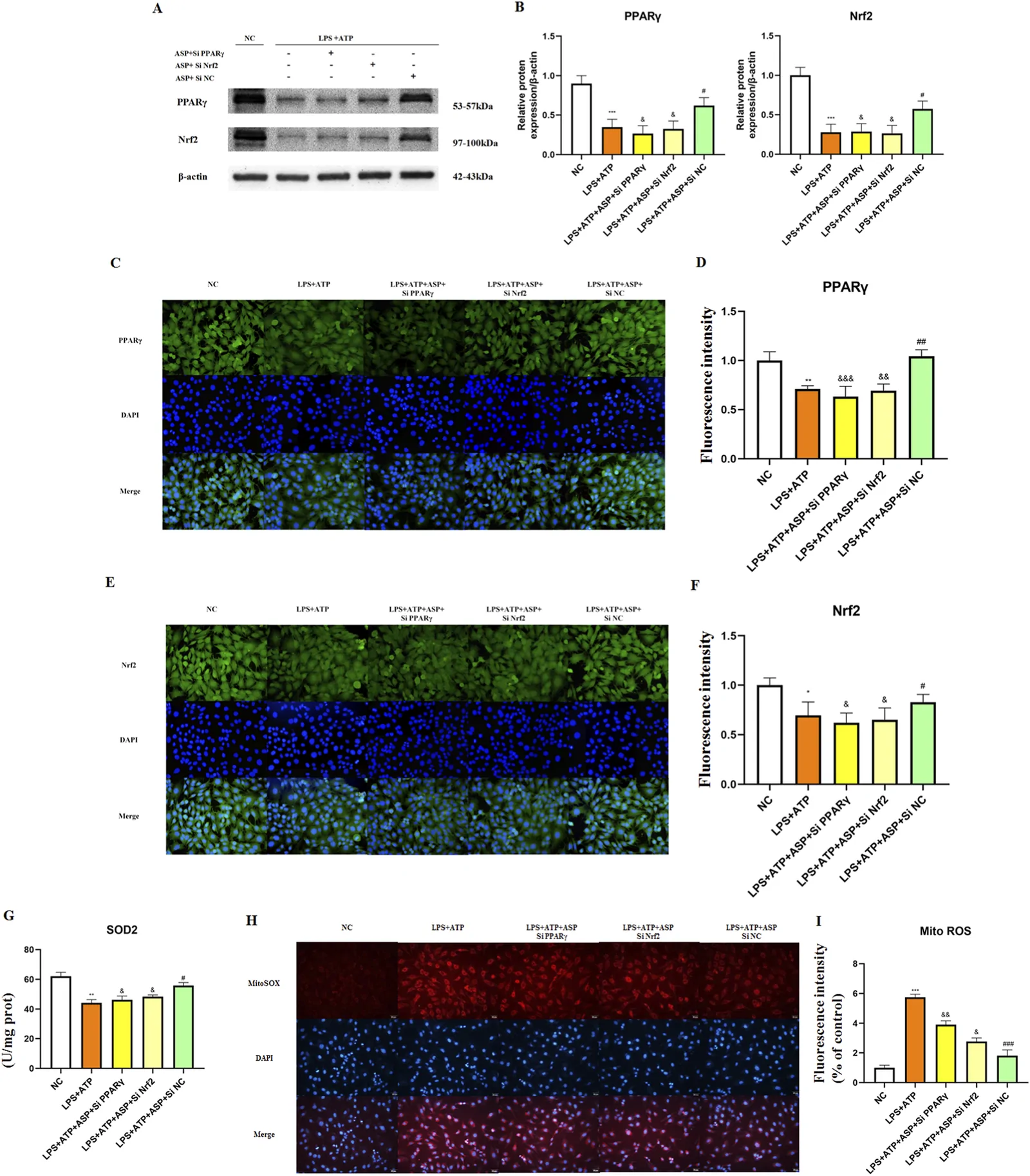
PPARγ and Nrf2 interact to inhibit mitochondrial ROS generation. **(A)** Western blot of PPARγ and Nrf2 after cell transfection. **(B)** WB quantification data. **(C)** Immunofluorescence of PPARγ after cell transfection. **(E)** Immunofluorescence of Nrf2 after cell transfection. **(D,F)** Fluorescence intensity quantification data. **(G)** SOD2 detection. **(H)** Fluorescence of mitochondrial ROS. **(I)** Fluorescence intensity quantification data. *p < 0.05, **p < 0.01, ***p < 0.001 versus NC. ^#^ *p* < 0.05, ^##^ *p* < 0.01, ^###^ *p* < 0.001 versus LPS + ATP. ^&^ *p* < 0.05, ^&&^ *p* < 0.01, ^&&&^ *p* < 0.001 versus Si NC.

Using the chemical agonists GW1929 and NK-252 was a positive verification method. We further knocked down the activities of PPARγ and Nrf2 through cell transfection, and re-validated the effects of PPARγ and Nrf2 on mitochondrial function from a reverse perspective. As shown in Figures 5A,B, when the PPARγ gene was blocked, the expression of both PPARγ and Nrf2 proteins was downregulated, and ASP could not exert its activating effect. This was consistent with the results after blocking the Nrf2 gene. Only when the PPARγ and Nrf2 genes were not interfered with could ASP exert its efficacy as an exogenous agonist. LPS + ATP affected the protein levels of PPARγ and Nrf2 rather than nuclear DNA, which was also the basis of ASP treatment. We also employed immunofluorescence detection to corroborate the conclusions drawn from WB. The results indicated that regardless of downregulating PPARγ or Nrf2 at the genetic level, the fluorescence intensity of both significantly decreased (Figures 5C–F). ASP failed to exert its original therapeutic function, which also reflected the treatment limitation of a natural drug.

When PPARγ or Nrf2 was knocked down, SOD2, as a downstream target gene, experienced significant inhibition of its transcriptional activation and a notable decrease in protein activity (Figure 5G). Consequently, it was unable to effectively eliminate mitochondrial ROS, leading to an increase in red fluorescence intensity (Figures 5H,I) and significantly affecting the therapeutic effect of ASP. Therefore, we suggested from a reverse perspective that there might be a synergistic effect between PPARγ and Nrf2, which influenced mitochondrial function through ROS.

### 3.7 Collaborative power of PPARγ and Nrf2 regulated pyroptosis

**FIGURE 6:**
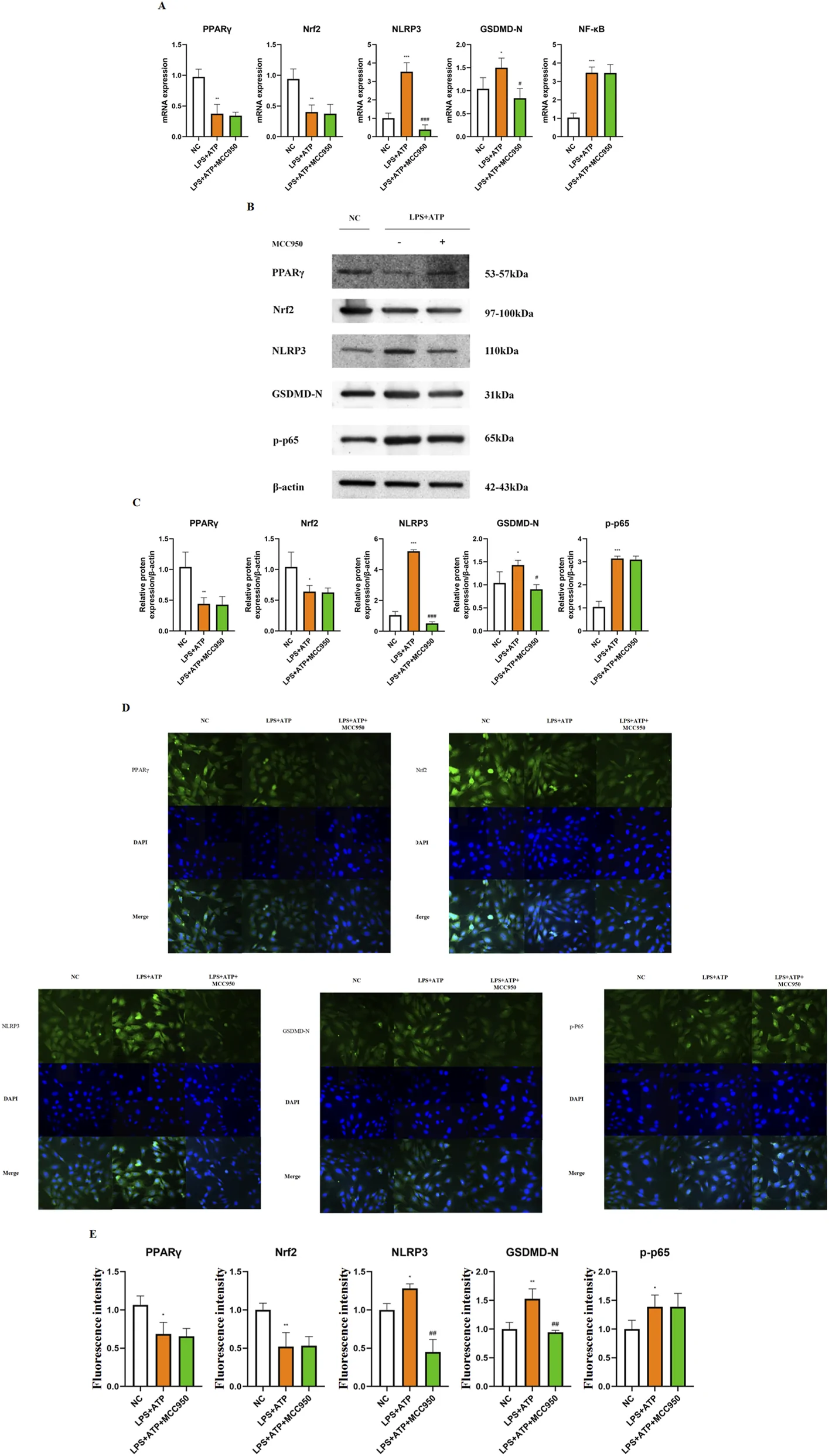
Collaborative power of PPARγ and Nrf2 regulates pyroptosis. **(A)** Treated ADTC5 cells were harvested, and total RNA was extracted, followed by qRT-PCR for detection of PPARγ, Nrf2, NLRP3, GSDMD-N, and NF-κB. **(B)** Treated ADTC5 cells were harvested, total proteins were extracted, and Western blot was used to detect the expression of PPARγ, Nrf2, NLRP3, GSDMD-N, and p-p65. **(C)** WB quantification data. **(D)** Immunofluorescence of PPARγ, Nrf2, NLRP3, GSDMD-N, and p-p65. **(E)** Fluorescence intensity quantification data. *p < 0.05, **p < 0.01, ***p < 0.001 versus NC. ^#^ *p* < 0.05, ^##^ *p* < 0.01, ^###^ *p* < 0.001 versus LPS + ATP.

**FIGURE 7:**
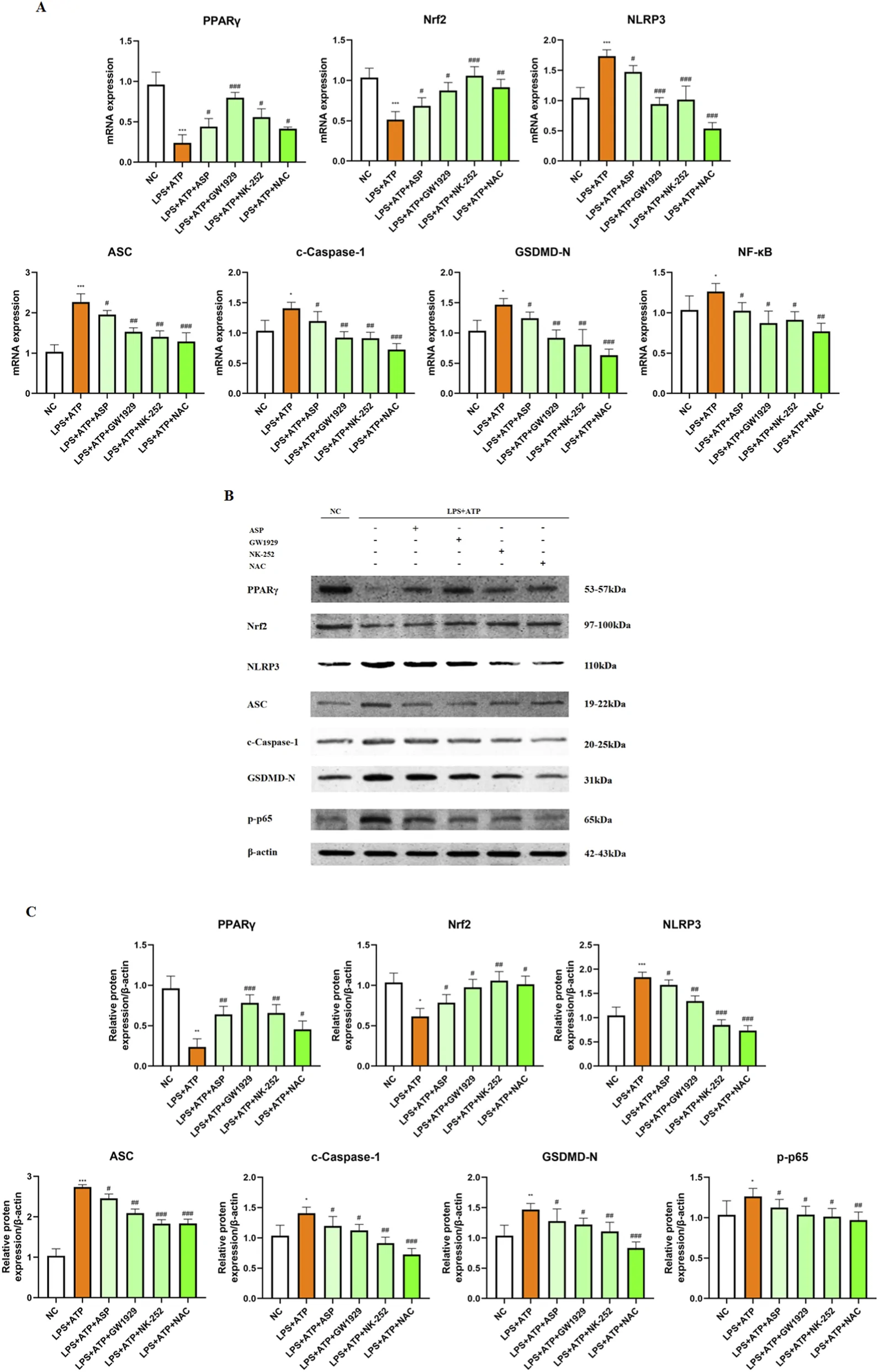
Pyroptosis interfered with the expressions of PPARγ and Nrf2 in chondrocytes. **(A)** Treated ADTC5 cells were harvested, and total RNA was extracted, followed by qRT-PCR for detection of PPARγ, Nrf2, NLRP3, ASC, c-Caspase-1, GSDMD-N, and NF-κB. **(B)** Treated ADTC5 cells were harvested, total proteins were extracted, and Western blot was used to detect the expression of PPARγ, Nrf2, NLRP3, ASC, c-Caspase-1, GSDMD-N, and p-p65. **(C)** WB quantification data. *p < 0.05, **p < 0.01, ***p < 0.001 versus NC. ^#^ *p* < 0.05, ^##^ *p* < 0.01, ^###^ *p* < 0.001 versus LPS + ATP.

To verify the relationship between PPARγ and Nrf2 and pyroptosis, we utilized the NLRP3 inhibitor MCC950. When MCC950 was used to intervene in ADTC5 cells, qRT-PCR and WB results showed no significant changes in PPARγ and Nrf2 at both the gene and protein levels, indicating that MCC950 did not affect them. However, both NLRP3 and GSDMD-N expressions were significantly downregulated, although there were no significant changes in the NF-κB gene and the p-p65 protein (Figures 6A–C). Immunofluorescence assay results showed no significant changes in the fluorescence intensity of PPARγ, Nrf2, and p-p65 after treatment with MCC950, while the fluorescence intensity of NLRP3 and GSDMD-N significantly decreased (Figures 6D,E). Based on this, we speculated that the NLRP3 pyroptosis pathway lay downstream of PPARγ and Nrf2. Combined with the results of Figure 7, it could be inferred that PPARγ and Nrf2 synergistically regulated pyroptosis.

### 3.8 ASP regulated pyroptosis and alleviated OA progression *in vivo* through the collaborative power of PPARγ and Nrf2

**FIGURE 8:**
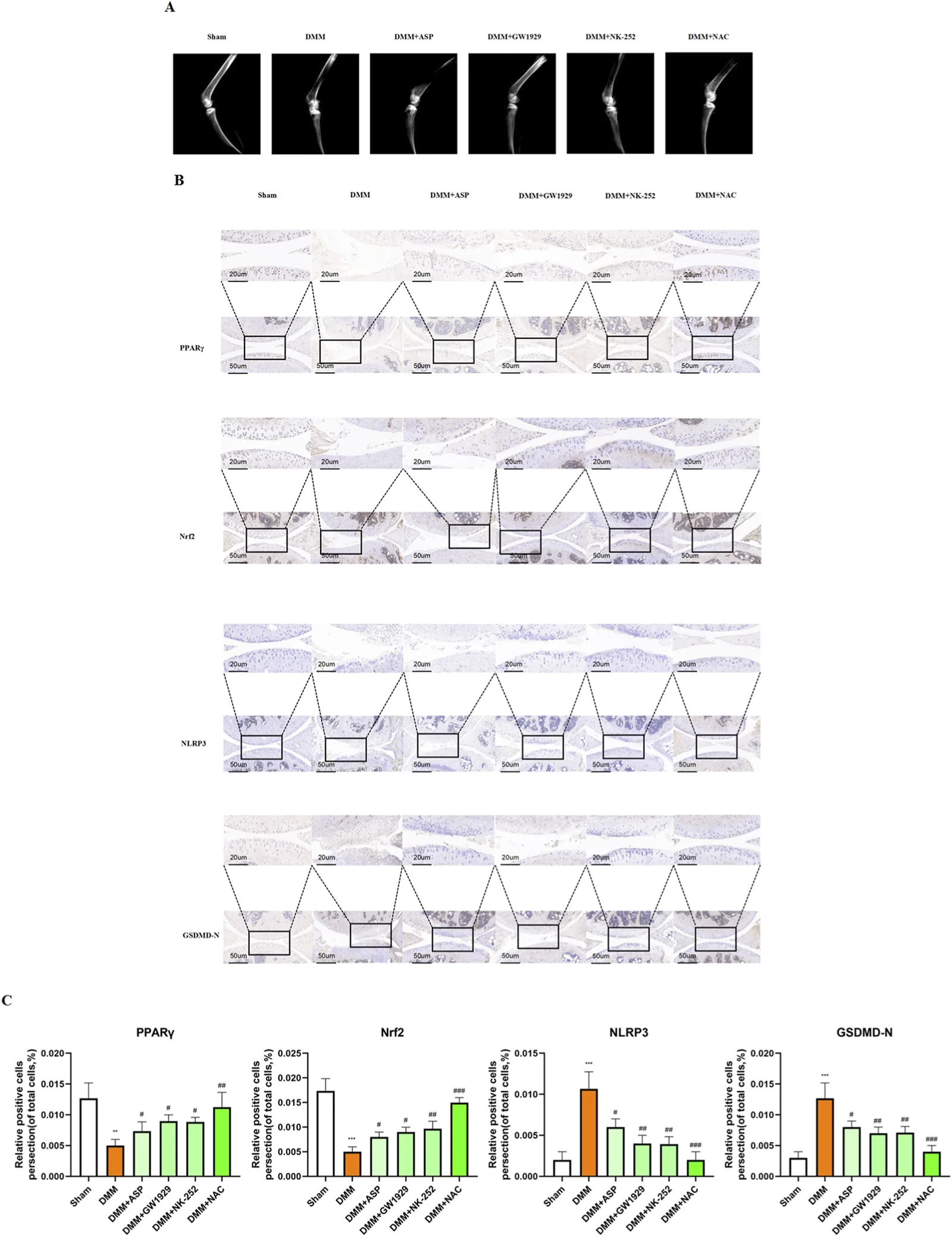
ASP regulated pyroptosis and alleviated OA progression *in vivo* through the collaborative power of PPARγ and Nrf2. **(A)** X-ray of the lower limb. **(B)** Immunohistochemistry of PPARγ, Nrf2, NLRP3, and GSDMD-N (×2.5 magnification, scale bar, 20 μm). **(C)** Immunohistochemistry quantification data. **p < 0.01, ***p < 0.001 versus Sham. ^#^ *p* < 0.05, ^##^ *p* < 0.01, ^###^ *p* < 0.001 versus DMM.

Based on cellular experiments, we conducted animal experiments, establishing a mouse OA model through DMM surgery and performing relevant tests after drug intervention. The lower limb X-ray demonstrated successful establishment of the OA model. Compared to the Sham group, mice in the DMM group exhibited joint space narrowing and osteophytosis. After ASP treatment, radiographic improvements were observed compared to the DMM group (Figure 8A). Immunohistochemistry revealed that compared to the Sham group, the expressions of PPARγ and Nrf2 decreased in the DMM group, while the expressions of NLRP3 and GSDMD-N increased, once again demonstrating that pyroptosis was involved in the pathogenesis of OA. Compared to the DMM group, ASP treatment could restore the protein levels of PPARγ and Nrf2, thereby inhibiting NLRP3 and GSDMD-N protein expressions (Figures 8B,C). Its effect was similar to that of GW1929 and NK-252, thus proving again that ASP could regulate pyroptosis through the collaborative power of PPARγ and Nrf2, thereby alleviating the progression of OA.

## 4 Discussion

OA is one of the most common joint diseases. It mainly damages articular cartilage, affects the whole joint, and ultimately causes joint dysfunction and even disability. The increased release of inflammatory cytokines during the occurrence and development of OA will lead to the metabolic disorder of chondrocytes and the degradation of ECM, further promoting the progression of the disease (Wojdasiewicz et al., 2014; Mobasheri et al., 2017).

Regulatory cell death has always been a research direction in the pathogenesis of OA, and pyroptosis, as one of the regulatory cell deaths, has gradually become an important topic since its report in 2001. It is characterized by the release of inflammatory cytokines and differs from other types of regulatory cell death. Pyroptosis is an important immune response for the body’s defense and plays a crucial role in resisting infection and activating endogenous danger signals (Bergsbaken et al., 2009). As the only cells of articular cartilage, chondrocytes can sense changes in the microenvironment inside the joint under physiological conditions, thereby maintaining a balance between ECM synthesis and degradation metabolism. However, in pathological conditions, chondrocytes experience cartilage degeneration due to regulatory cell death, leading to a decrease in their number and triggering OA. Previous studies have suggested that cell apoptosis is an important mechanism leading to the progression of OA. However, recent studies have found that apoptotic cells do not promote an inflammatory response. Only through pyroptosis can pro-inflammatory cytokines be secreted to accelerate OA progression (Berenbaum and Walker, 2020; An et al., 2020; Zu et al., 2019).

The canonical pyroptosis is an inflammatory death mode highly dependent on the activation of caspase-1. It is widely involved in the process of innate immunity and acquired immune system resistance to microbial invasion by activating a variety of protein complexes (Man and Kanneganti, 2015). The inflammasome is a wheel-like structure with extensive activity existing in the cytoplasm, which is mainly composed of three parts: receptor protein, ASC as an adaptor protein, and caspase-1 as an effector protein. After activation, caspase-1 can further cleave and activate IL-18 and IL-1β and cleave the N-terminal sequence of GSDMD. The latter binds to the membrane to produce membrane pores, leading to pyroptosis (Sborgi et al., 2016; He et al., 2015).

Our experimental specimens were obtained from the tibial plateau of OA patients who underwent total knee arthroplasty. We regarded the lateral platform as relatively normal cartilage (NC) and the medial platform as OA-damaged cartilage. The gross appearance of the specimens, HE staining, and safranin O staining showed significant differences between the two groups, which were further verified through OARSI and Mankin scores. We conducted immunohistochemistry and WB on cartilages of both groups, and the results showed that the expression of NLRP3 inflammasome was increased, and GSDMD-N was upregulated, but the expressions of PPARγ, Nrf2, and SOD2 were downregulated. The levels of IL-1β and IL-18 in the serum of OA patients were higher than those of the normal control, which showed that NLRP3 inflammasome-mediated pyroptosis was involved in the pathogenesis of OA. SOD2 is located in mitochondria to selectively clear mitochondrial ROS. We have previously shown that the PPARγ/SOD2/ROS signaling axis could regulate oxidative stress in OA chondrocytes. This article continued to explore its relationship with pyroptosis.

To support the conclusion of clinical specimen testing, we proceeded with cellular experiments. By establishing a pyroptosis model of chondrocytes, we showed that pyroptosis negatively regulated chondrocyte activity and increased cytotoxicity and cell death. Oxidative stress is a pathological state caused by the excessive production of ROS. The results also showed that mitochondrial ROS showed an upward trend in chondrocytes undergoing pyroptosis, and the reason for this was the decrease in SOD2 activity. ROS participates in the activation of the NLRP3 inflammasome and induces canonical pyroptosis of chondrocytes (Sho and Xu, 2019). Chondrocytes with pyroptosis continue to swell until the cell membrane breaks, leading to the release of cell contents, thereby activating a strong inflammatory response (Liu et al., 2016).

ASP is a traditional Chinese medicine extract with antioxidant and anti-inflammatory activities. It has certain prospects in the treatment of OA, but its specific mechanism must be elucidated. In this experiment, the trend of pyroptosis decreased, and chondrocyte viability was restored after ASP pretreatment. Further results revealed that ASP cleared mitochondrial ROS and inhibited pyroptosis by upregulating SOD2 activity. Therefore, we showed that mitochondrial ROS was associated with pyroptosis. However, there was no literature on the regulatory effect and related mechanisms of ASP on pyroptosis. We inferred from the results of positive control groups that the regulatory relationship between ASP and pyroptosis was related to PPARγ and Nrf2.

In order to control the level of ROS and prevent its accumulation, human cells have formed a complex antioxidant defense system (Khan et al., 2018; Ye et al., 2014). Among them, Nrf2 is an important redox-sensitive transcription factor, which is conducive to improving the oxidative stress state of the body, promoting cell survival, and maintaining redox homeostasis by regulating the expression of phase II antioxidant enzymes (Oh et al., 2019; Pan et al., 2019). PPARγ is a ligand-activated nuclear receptor that plays an important regulatory role in the process of glucose and lipid metabolism and is the main transcription factor of energy metabolism (Grygiel-Gorniak, 2014). Recently, the regulatory role of PPARγ in the inflammatory process is receiving increasing attention. OA is cartilage degeneration caused by inflammation, leading to chondrocyte death and ECM degradation. PPARγ can exert anti-inflammatory effects by regulating the transcription and translation of target genes, antagonizing the NF-κB inflammatory signaling pathway, reducing the expression of inflammatory cytokines, and regulating the inflammatory process (Chen et al., 2015; Afif et al., 2007).

In our experiment, the expressions of PPARγ and Nrf2 were downregulated in OA cartilage and were also inhibited in the ADTC5 cells induced by LPS + ATP, indicating that PPARγ and Nrf2 were involved in pyroptosis. To determine the relationship between PPARγ and Nrf2, as well as their regulatory relationship with pyroptosis, we performed cell transfection to knock down the expression of PPARγ or Nrf2. In addition, we intervened with an NLRP3 inhibitor, and the results indicated the collaborative power between PPARγ and Nrf2, jointly regulating pyroptosis, which was consistent with previous literature (Polvani et al., 2012). In cellular experiments, ASP could activate SOD2 transcription and synthesis, clear mitochondrial ROS, and stabilize MMP, so as to downregulate pyroptosis. This downregulation must be achieved through PPARγ and Nrf2 because when PPARγ or Nrf2 was knocked down, ASP was unable to exert its effect as an exogenous agonist. In animal experiments, the imaging and immunohistochemical detection results also showed the involvement of pyroptosis in OA pathogenesis, and ASP inhibited pyroptosis by improving PPARγ and Nrf2 activity, thereby alleviating OA progression.

**FIGURE 9:**
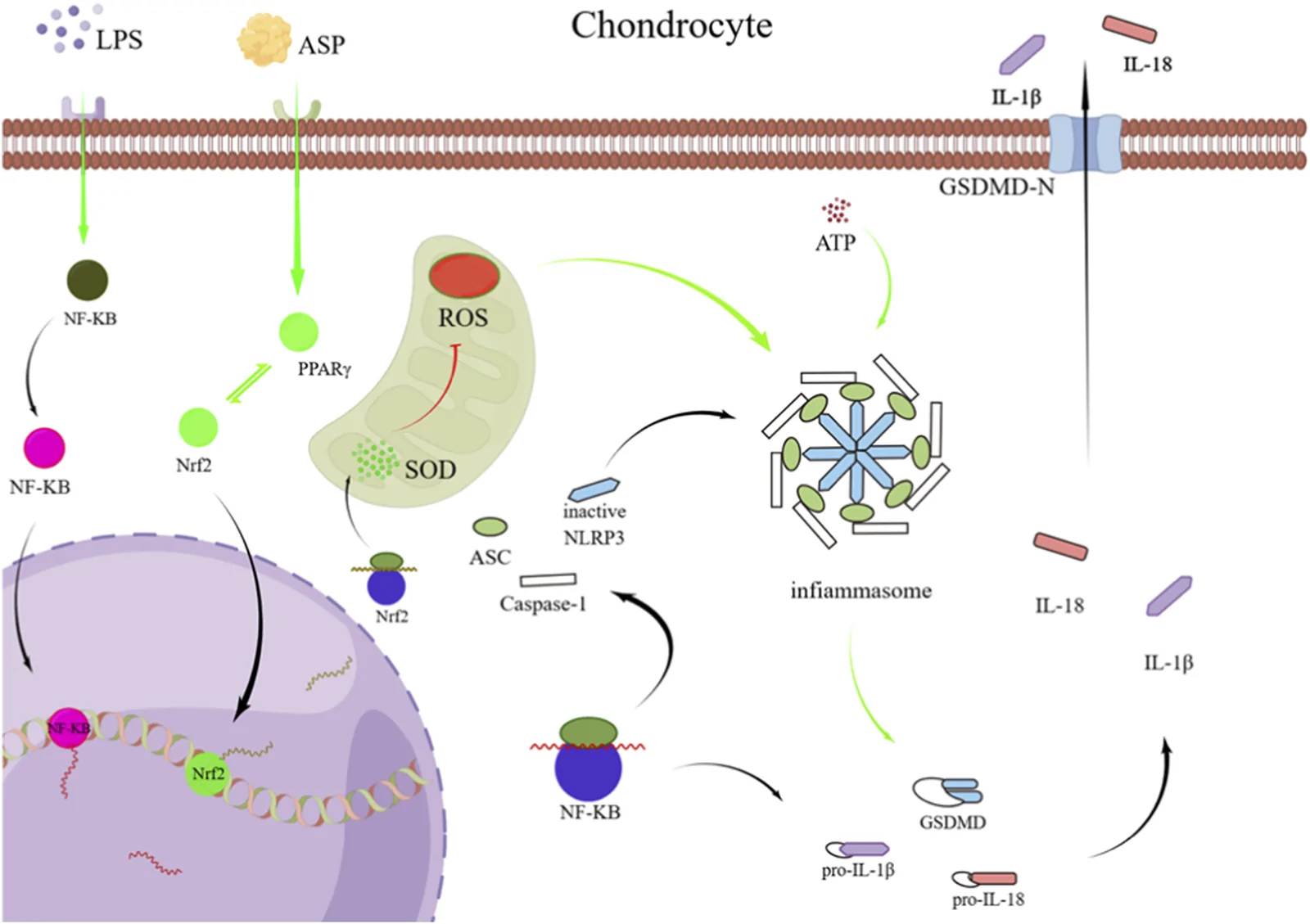
Our proposed hypothesis about the mechanisms of how ASP improves the collaborative power of PPARγ and Nrf2 in inhibiting NLRP3 inflammasome activation and alleviating pyroptosis in OA.

In the pathogenesis of OA, the expressions of PPARγ and Nrf2 decreased, but the NF-κB signaling pathway was activated, which regulated NLRP3 transcription and pro-IL-1β expression, contributing to the priming of inflammasome components, while NLRP3 activation and caspase-1 cleavage execute pyroptosis. ASP intervention could improve the collaborative power of PPARγ and Nrf2, which act as a modulator of oxidative stress pathways and upstream regulator of antioxidant defense systems to exert antioxidant and anti-inflammatory effects, downregulate the NF-κB signaling pathway, inhibit NLRP3 inflammasome synthesis, reduce the release of IL-1β and IL-18, and alleviate cartilage degeneration (Figure 9). This would update our understanding of the molecular mechanism of OA and provide a new drug for treatment. However, the direct molecular target of ASP remains unidentified.

## 5 Limitations

(1) Biological limitation: Single OA model (DMM) does not capture human OA heterogeneity because the chondrocyte monoculture lacks joint microenvironment complexity. (2) Mechanistic limitations: No direct binding assays for ASP and no rescue experiments fully validating pathway hierarchy. (3) Translational limitations: ASP dosing relevance in humans is unknown. Pharmacokinetics and bioavailability were not assessed. (4) Pathway limitations: Other OA pathways were not explored. (5) We did not perform related experiments about ASP alone on normal chondrocytes in this study to rule out potential off-target or basal effects of ASP on PPARγ/Nrf2 signaling. (6) Synergy between PPARγ and Nrf2 was based on reciprocal changes in expression when the other is knocked down, which did not prove direct interaction. (7) Only one dose of ASP and one timepoint were used in the DMM model. A dose–response study and longer follow-up would strengthen translational relevance as well as provide joint pain and behavioral data.

## Data Availability

The raw data supporting the conclusions of this article will be made available by the authors, without undue reservation.
